# Normative 3D gait data of healthy adults walking at three different speeds on an instrumented treadmill in virtual reality

**DOI:** 10.1016/j.dib.2024.110230

**Published:** 2024-02-22

**Authors:** Rachel Senden, Rik Marcellis, Paul Willems, Marianne Witlox, Kenneth Meijer

**Affiliations:** a Department of Physical Therapy, Maastricht University Medical Center (MUMC+), Maastricht, the Netherlands; b Department of Nutrition and Movement Sciences, NUTRIM School of Nutrition and Translational Research in Metabolism, Maastricht University, Maastricht, the Netherlands; c Department of Orthopaedic Surgery, MUMC+, Maastricht, the Netherlands; d Research School CAPHRI, Maastricht University, Maastricht, the Netherlands

**Keywords:** Gait analysis, Healthy adults, CAREN, Spatiotemporal parameters, Joint angles, Ground reaction forces, Joint moments, Joint powers

## Abstract

A normative gait dataset of 246 healthy adults (122 men / 124 women, range in age 18-91 years, body weight 46.80-116.10 kg, height 1.53-1.97 m and BMI 18.25-35.63 kg/m2) is presented and publicly shared for three walking speed conditions.

Raw and processed data are presented for each subject separately and for each walking speed, including data of every single step of both legs. The subject demographics and results from the physical examination are also presented which allows researchers and clinicians to create a self-selected reference group based on specific demographics. Besides the data per individual, data are also presented in age and gender groups. This provides a quick overview of healthy gait parameters which is relevant for use in clinical practice.

Three dimensional gait analysis was performed at the Computer Assisted Rehabilitation Environment (CAREN) at the Maastricht University Medical Centre (MUMC+). Subjects walked on the instrumented treadmill surrounded with twelve 3D cameras, three 2D cameras and a virtual industrial environment projected on a 180° screen using the Human Body Lower Limb Model with trunk markers (HBM-II) as biomechanical model [1], [2].

Subjects walked at comfortable walking speed, 30% slower and 30% faster. These walking speed conditions were applied in a random sequence. Comfortable walking speed was determined using a RAMP protocol: subjects started to walk at 0.5m/s and every second the speed was increased with 0.01 m/s until the preferred speed was reached. The average of three repetitions was considered the comfortable speed. For each walking speed condition, 250 steps were recorded.

The 3D gait data was collected using the D-flow CAREN software. For each subject, raw data of each walking speed condition is provided in .mox files, including the output from the model such as subject data (e.g. gender, body mass, knee and ankle width), center of mass (CoM), marker and force data, kinematic data (joint angles) and kinetic data (joint moments, ground reaction forces (GRFs) and joint powers) for each single step of both legs. Unfiltered and filtered data are included. C3D files with raw marker and GRF data were recorded in Nexus (Vicon software, version 2.8.1) and are available upon request.

Raw data were processed in Matlab (Mathworks 2016), including quality check, step determination and the exportation of data to .xls files. For each adult and for each walking speed, an .xls file was created, containing spatiotemporal parameters, medio-lateral (ML) and back-forward (BF) margins of stability (MoS), 3D joint angles, anterior-posterior (AP) and vertical GRFs, 3D joint moments and 3D joint power of each step of both legs. Overview files per walking speed condition are created in .xls, presenting the averaged gait parameters (calculated as average over all valid steps) of every subject. The processed data is also presented and visualized per gender for different age groups (18-29 years, 30-39 years, 40-49 years, 50-59 years, 60-69 years, ≥70 years). This can serve as normative data for treadmill based 3D gait analyses in adults, applicable for clinical and research purposes. Data is available at OSF.io (https://osf.io/t72cw/).

Specifications Table

**Table:** 

Subject	Walking biomechanics.
Specific subject area	3D treadmill based gait analysis in 246 healthy adults, aged 18-91 years walking at comfortable, slow and fast walking speed in virtual reality.
Type of data	**Raw data:** For each subject, the .mox files of each walking speed condition are presented. This includes the output of the Human Body Lower Limb Model with trunk markers (HBM-II) such as subject, marker, force, kinematic and kinetic data for each single step of the left and right leg. Unfiltered and filtered data are included.
	**Processed data:** *Individual .xls files:* For each subject, an .xls file was created per walking speed condition presenting spatiotemporal parameters, medio-lateral (ML) and back-forward (BF) Margins of Stability (MoS), 3D joint angles, vertical and anterior-posterior (AP) ground reaction forces (GRF), 3D joint moments, and 3D joint power of every single step for the left and right leg. *Overview .xls files:* An overview file per walking speed condition was created, containing the averaged gait parameters of every subject for the left and right leg. The averaged gait parameters were calculated as the average over all valid steps. *AgeGender Group .xls files:* For clinical application, gait parameters of both legs are also presented per gender for the different age groups (18-29 years, 30-39 years, 40-49 years, 50-59 years, 60-69 years and ≥70 years). Table: For each walking speed condition, spatiotemporal parameters (for both left and right leg), ML and BF MoS are presented per gender and age groups in a table. Figure: For each walking speed condition, 3D joint angles, vertical and AP GRFs, 3D joint moments and 3D joint power of the right leg are visualized per gender and age group.
How the data were acquired	CAREN (MotekForce Link) with HBM-II comprising a dual belt instrumented treadmill (force plates 1000Hz), 12 motion capture cameras (100Hz, Vicon), 3 2D cameras and a virtual reality was used. A subject calibration was done, after which the comfortable walking speed was determined, followed by a six minute familiarization trial. Next, 250 steps were recorded at comfortable, slow (-30%) and fast (+30%) walking speed. The Vicon Nexus software (V2.8.1) controlled the 3D motion capture system. The Motek D-flow software (V3.28.0) controlled hardware components of CAREN. Custom-made algorithms programmed in Matlab (Mathworks 2016) were used for data quality check, step detection and export of data.
Data format	Raw Analyzed Filtered
Description of data collection	246 Healthy adults walked at comfortable, slow and fast speed. Adults were ambulant, willing to participate, ≥18 years and had no muscular, skeletal or cardiopulmonary disorders and received no medical interventions influencing gait. Raw and processed data were collected for each adult. Data is also presented and visualized in age and gender groups. Joint angles, GRFs, joint moments and power were time normalized (0-100%). GRF were normalized for weight.
Data source location	Institution: Maastricht University Medical Centre (MUMC+) City/Town/Region: Maastricht Country: The Netherlands
Data accessibility	Repository name: OSF.io Direct URL to data: https://osf.io/t72cw/ Identifier: DOI 10.17605/OSF.IO/T72CW
Related research article	Not applicable

## 1 Value of the Data

• This gait dataset of healthy adults can serve as normative data for clinical and research purposes. The known influence of demographics [3] and walking speed [4] on gait can be eliminated by selecting a matched control group to compare the patients gait with. This ensures a correct interpretation of pathological gait.
• This data can be used to explore walking biomechanics in healthy adults aged 18 to 91 years. E.g. the effect of subject demographics on spatiotemporal parameters, kinematics and kinetics can be investigated.
• This dataset allows to explore the effect of walking speed on gait parameters, since gait data is available for three different walking speeds.
• Since data of many successive steps is available for each subject and each walking speed condition, this data allows to further investigate dynamic stability and variability in gait at different walking speeds.
• The data can be useful for any scientist interested in gait and can be used for any scientific purposes in case permission is granted.

## 2 Objective

To create a normative gait dataset for treadmill walking in a virtual environment at various walking speeds in a group of healthy adults.

## 3 Data Description

**File 01_Demo_PhysEx.xlsx**: contains demographic data including age (years), sex (M/F), body mass (kg), height (m), leg length (m) and age group (18-29 years, 30-39 years, 40-49 years, 50-59 years, 60-69 years, ≥70 years). In addition, the results of the physical examination are presented per adult.

**File 02_Overview_comf.xlsx**: contains the averaged walking biomechanics (calculated over all valid strides) for each adult walking at comfortable speed. Spatiotemporal parameters, medio-lateral (ML) and back-forward (BF) Margins of Stability (MoS), 3D joint angles, vertical and anterior-posterior (AP) ground reaction forces (GRF), 3D joint moments and 3D joint power are included for the left and right leg. This data is based on the individual excel files that can be found in “Folder 27_xls files”. The description of the parameters can be found in File 28_Description_parameters.

**File 03_Overview_fast.xlsx:** contains the averaged walking biomechanics (calculated over all valid strides) for each adult walking at fast speed (comfortable +30%). Spatiotemporal parameters, ML and BF MoS, 3D joint angles, vertical and AP GRF, 3D joint moments and 3D joint power are included for the left and right leg. This data is based on the individual excel files that can be found in “Folder 27_xls files”. The description of the parameters can be found in File 28_Description_parameters.

**File 04_Overview_slow.xlsx:** contains the averaged walking biomechanics (calculated over all valid strides) for each adult walking at slow speed (comfortable -30%). Spatiotemporal parameters, ML and BF MoS, 3D joint angles, vertical and AP GRF, 3D joint moments and 3D joint power are included for the left and right leg. This data is based on the individual excel files that can be found in “Folder 27_xls files”. The description of the parameters can be found in File 28_Description_parameters.

**File 05_AgeGenderGroup_comf.xlsx:** contains walking biomechanics categorized per gender and age group (18-29 years, 30-39 years, 40-49 years, 50-59 years, 60-69 years and ≥70 years) walking at comfortable speed. Group averages and standard deviations are included for spatiotemporal parameters, ML and BF MoS, 3D joint angles, vertical and AP GRF, 3D joint moments and 3D joint power for the left and right leg. The description of the parameters can be found in File 28_Description_parameters.

**File 06_AgeGenderGroup_fast.xlsx**: contains walking biomechanics categorized per gender and age group (18-29 years, 30-39 years, 40-49 years, 50-59 years, 60-69 years and ≥70 years) walking at fast speed (comfortable -30%). Group averages and standard deviations are included for spatiotemporal parameters, ML and BF MoS, 3D joint angles, vertical and AP GRF, 3D joint moments and 3D joint power for the left and right leg. The description of the parameters can be found in File 28_Description_parameters.

**File 07_AgeGenderGroup_slow.xlsx:** contains walking biomechanics categorized per gender and age group (18-29 years, 30-39 years, 40-49 years, 50-59 years, 60-69 years and ≥70 years) walking at slow speed (comfortable -30%). Group averages and standard deviations are included for spatiotemporal parameters, ML and BF MoS, 3D joint angles, vertical and AP GRF, 3D joint moments and 3D joint power for the left and right leg. The description of the parameters can be found in File 28_Description_parameters.

**File 08_Table_Spatiotemporal_comf.png**: represents spatiotemporal parameters per gender and age group (18-29 years, 30-39 years, 40-49 years, 50-59 years, 60-69 years and ≥70 years), including group averages and standard deviations for the left and right leg separately, for walking at comfortable speed.

**File 09_Fig_JointAngles_comf.pdf:** represents the averaged 3D joint angle waveforms of the right leg, normalized for time (0-100%), per gender and age group (18-29 years, 30-39 years, 40-49 years, 50-59 years, 60-69 years and ≥70 years), for walking at comfortable speed.

**File 10_Fig_GRF_comf.pdf:** represents the averaged vertical and AP GRF waveforms of the right leg, normalized for time (0-100%), per gender and age group (18-29 years, 30-39 years, 40-49 years, 50-59 years, 60-69 years and ≥70 years) for walking at comfortable speed. GRFs were normalized for body weight according to Hof [5].

**File 11_Fig_JointMoments_comf.pdf:** represents the averaged 3D joint moment waveforms of the right leg, normalized for time (0-100%), per gender and age group (18-29 years, 30-39 years, 40-49 years, 50-59 years, 60-69 years and ≥70 years) for walking at comfortable speed.

**File 12_Fig_JointPower_comf.pdf:** represents the averaged 3D joint power waveforms of the right leg, normalized for time (0-100%), per gender and age group (18-29 years, 30-39 years, 40-49 years, 50-59 years, 60-69 years and ≥70 years) for walking at comfortable speed.

**File 13_Table_MoS_comf.png:** represents the averaged ML and BF MoS per gender and age group (18-29 years, 30-39 years, 40-49 years, 50-59 years, 60-69 years and ≥70 years), for walking at comfortable speed.

**File 14_Table_Spatiotemporal_fast.png**: represents spatiotemporal parameters per gender and age group (18-29 years, 30-39 years, 40-49 years, 50-59 years, 60-69 years and ≥70 years), including group averages and standard deviations for the left and right leg separately, for walking at fast (comfortable +30%) speed.

**File 15_Fig_JointAngles_fast.pdf:** represents the averaged 3D joint angle waveforms of the right leg, normalized for time (0-100%), per gender and age group (18-29 years, 30-39 years, 40-49 years, 50-59 years, 60-69 years and ≥70 years), for walking at fast (comfortable +30%) speed.

**File 16_Fig_GRF_fast.pfd:** represents the averaged vertical and AP GRF waveforms of the right leg, normalized for time (0-100%), per gender and age group (18-29 years, 30-39 years, 40-49 years, 50-59 years, 60-69 years and ≥70 years) for walking at fast (comfortable +30%) speed. GRFs were normalized for body weight according to Hof [5].

**File 17_Fig_JointMoments_fast.pdf:** represents the averaged 3D joint moment waveforms of the right leg, normalized for time (0-100%), per gender and age group (18-29 years, 30-39 years, 40-49 years, 50-59 years, 60-69 years and ≥70 years) for walking at fast (comfortable +30%) speed.

**File 18_Fig_JointPower_fast.pdf:** represents the averaged 3D joint power waveforms of the right leg, normalized for time (0-100%), per gender and age group (18-29 years, 30-39 years, 40-49 years, 50-59 years, 60-69 years and ≥70 years) for walking at fast (comfortable +30%) speed.

**File 19_Table_MoS_fast.png:** represents the averaged ML and BF MoS per gender and age group (18-29 years, 30-39 years, 40-49 years, 50-59 years, 60-69 years and ≥70 years), for walking at fast (comfortable +30%) speed.

**File 20_Table_Spatiotemporal_slow.png**: represents spatiotemporal parameters per gender and age group (18-29 years, 30-39 years, 40-49 years, 50-59 years, 60-69 years and ≥70 years), including group averages and standard deviations for the left and right leg separately, for walking at slow (comfortable -30%) speed.

**File 21_Fig_JointAngles_ slow.pdf:** represents the averaged 3D joint angle waveforms of the right leg, normalized for time (0-100%), per gender and age group (18-29 years, 30-39 years, 40-49 years, 50-59 years, 60-69 years and ≥70 years), for walking at slow (comfortable -30%) speed.

**File 22_Fig_GRF_slow.pdf:** represents the averaged vertical and AP GRF waveforms of the right leg, normalized for time (0-100%), per gender and age group (18-29 years, 30-39 years, 40-49 years, 50-59 years, 60-69 years and ≥70 years) for walking at slow (comfortable -30%) speed. GRFs were normalized for body weight according to Hof [5].

**File 23_Fig_JointMoments_slow.pdf:** represents the averaged 3D joint moment waveforms of the right leg, normalized for time (0-100%), per gender and age group (18-29 years, 30- 39 years, 40-49 years, 50-59 years, 60-69 years and ≥70 years) for walking at slow (comfortable -30%) speed.

**File 24_Fig_JointPower_slow.pdf:** represents the averaged 3D joint power waveforms of the right leg, normalized for time (0-100%), per gender and age group (18-29 years, 30-39 years, 40-49 years, 50-59 years, 60-69 years and ≥70 years) for walking at slow (comfortable -30%) speed.

**File 25_Table_MoS_slow.png:** represents the averaged ML and BF MoS per gender and age group (18-29 years, 30-39 years, 40-49 years, 50-59 years, 60-69 years and ≥70 years), for walking at slow (comfortable -30%) speed.

**Folder 26_mox files**: contains the .mox files for each adult, organized per walking speed. The.mox files contain subject data (e.g. gender, body mass, knee and ankle width), marker position and force plate data, kinematic data (joint angles), kinetic data (GRF, joint moment, joint power) generated by CAREN software (D-flow).

**Folder 27_xls files:** contains the processed data for each adult, containing spatiotemporal parameters, MoS, joint angles, GRF, joint moments, joint power including every valid step of both legs, organized per walking speed.

**File 28_Description parameters**: contains information for interpreting .xls processed gait data. The layout and parameters are described in detail.

## 4 Experimental Design, Materials and Methods

### 4.1 CAREN system

Three dimensional gait analysis was performed at the Computer Assisted Rehabilitation Environment (CAREN, Motek Medical BV, Amsterdam, The Netherlands) at the MUMC+. CAREN is an advanced system for human movement assessment, including a dual-belt instrumented treadmill (ForceLink, Culemborg, The Netherlands) mounted on a 6 degrees-of-freedom movable platform with a 12 camera 3D motion capture system (Vicon Nexus V2.8.1, Oxford Metrics Groups, Oxford, UK, 100 Hz) and an immersive (industrial) environment that is projected on a 180° cyclindric screen. The human body lower limb model with trunk markers (HBM-II) with real-time biomechanics facilitates the analysis of spatiotemporal parameters, joint kinematics and kinetics. The individual treadmill belts have a length and width of 2.15 ×0.5 m, a motor of 6.28 kW per belt, a belt speed update frequency of 60 Hz, and a speed range of 0-18 km/h, 1000 Hz). In addition, three 2D cameras are used to record the sagittal and frontal view. Dedicated real-time Motek software (D-Flow version 3.28.0) is used to control the hardware components of CAREN (e.g. projectors, platform, treadmill). The Nexus software of Vicon (version V2.8.1) is used to control the 3D motion capture system.

### 4.2 System preparation and settings

In preparation for the measurement, the 3D motion capture cameras are calibrated using Nexus V2.8.1, according to the dynamic calibration procedure embedded in Vicon, as described in the CAREN user manual (Motek Medical BV, December 8, 2021). The force plates are zero leveled using Nexus V2.8.1. Force threshold for step detection in Nexus was set at 25 N. For the inverse dynamics, a filter on marker and force plate data of 6 Hz is used. The force plate configurations for the analog data was set at 0 Hz for the low-pass prefilter frequency and 20 N for the force threshold. All filters were unidirectional 2nd order Butterworth filters.

The Gait Graphs_HBM2.caren application in D-flow, including the projection of a virtual industrial environment, was used for gait analysis of each adult.

The Nexus software of Vicon (version 2.8.1) and D-flow Motek software (version 3.28.0) were continuously connected to each other. No markers or stick figure were visualized during the measurements.

### 4.3 Subject preparation

1. Each adult underwent a standardized physical examination according to Becher et al. [6], conducted by two experienced operators (RS, RM). The physical examination included passive and active range of motion (°) of hip, knee and ankle, strength and balance measurements. Also body height (m) and leg length (m, spina iliaca anterior superior to medial malleolus) were measured.
2. HBM-II [1] was used as biomechanical model. Therefore twenty-six reflective markers were attached to specific bony landmarks at the skin of the adult by two operators (RM, RS).
3. A safety harness was put on with which the adult was secured to the system to prevent falling.
4. A static and dynamic subject calibration was conducted and recorded in Nexus V2.8.1. The adult had to stand for 5 seconds in T-pose (feet at shoulder width and parallel to the Z-axis of the coordinate system in D-flow, each foot positioned at one belt and arms spread at shoulder height), followed by a few steps of walking at a random speed. This was recorded and directly processed in Nexus (V2.8.1).
5. The static calibration (T-pose) was repeated (step 4) and now recorded in D-flow. This resulted in body weight (kg), knee and ankle width (mm) for the left and right leg individually.
6. The medial markers were removed, and a RAMP protocol was started to determine the comfortable walking speed. The adult started to walk at 0.5 m/s and every second, the speed was gradual increased with 0.01 m/s. The adult was asked to indicate when the comfortable speed, defined as the preferred speed one would assume when walking outside, was reached. This was repeated three times of which the average was considered the comfortable walking speed.
7. A familiarization trial of 6 minutes walking at comfortable speed followed, to ensure that normal gait pattern was adopted [7].

### 4.4 Measurement

The measurements consisted of walking in the virtual industrial environment at comfortable, slow (30% slower than comfortable) and fast (30% faster than comfortable) speed. The sequence of the walking speed conditions was randomly applied and varied between subjects. For each walking speed condition, 250 steps were recorded in D-flow (version 3.28.0) and Nexus (V2.8.1).

Measurements were performed in underwear and with standardized gymnastic shoes to eliminate the effect of shoe wear on gait. A safety harness was worn to prevent falling.

### 4.5 Data analysis

Raw data files (.mox) were obtained by D-flow and were loaded into Matlab (R2016a, Mathworks, Natick, MA, USA). Custom-made algorithms were used to check the quality of the data, including for instance checking the visibility of all markers. Gaps in marker detection <30 samples were interpolated using cubic spline fitting, while gaps >30 samples were identified and removed. Moreover, it was checked whether both force plates were hit by one leg. Steps where both feet hit one belt were excluded for kinetic analysis.

The custom-made algorithms programmed in Matlab (R2016a, Mathworks, Natick, MA, USA) were also used to identify steps. Step detection is based on a combination of heel marker kinematics and force plate data (exceeding the threshold of 50 N) as described by Zeni et al. [8]. The events calculated with the marker method, are corrected with the mean difference of the force threshold events, calculated for those steps with the feet on different platforms. If the standard deviation of the difference of one event exceeded the value of 2.5 samples or when there are less than five good left and right good kinematic steps, the trial is excluded. The mean standard deviation was 0.69 +/- 0.19 sample for all events, thus this difference is very regular for normal walking.

Gait parameters were calculated using the custom-made Matlab scripts, relying on published formulas and principles.

### 4.6 Gait parameters

The following gait parameters were calculated and exported to .xls files (the exact calculation of the parameters is described in file ‘28_Description_parameters’):

- Spatiotemporal parameters such as step length (m) and step time (s).
- Joint angles of trunk, pelvis, hip, knee and ankle in the sagittal, frontal and transversal plane, presented as a function of the gait cycle (0-100%). Data is time normalized with 0% representing initial contact and 100% representing next initial contact of the same leg.
- Vertical and AP GRF presented as a function of the gait cycle (0-100%). Data is time normalized with 0% representing initial contact and 100% representing next initial contact of the same leg. GRF is normalized for weight according to Hof et al. [5].
- Joint moments of trunk, hip, knee and ankle in the sagittal, frontal and transversal plane, presented as a function of the gait cycle (0-100%). Data is time normalized with 0% representing initial contact and 100% representing next initial contact of the same leg.
- 3D joint power of trunk, hip, knee and ankle, presented as a function of the gait cycle (0-100%). Data is time normalized with 0% representing initial contact and 100% representing next initial contact of the same leg.
- MoS in ML and BF direction. MoS is a measure for dynamic stability and is calculated using the definition of Hof et al. [9]. Base of support is calculated using the center of pressure and the center of mass as determined based on HBM-II. MoS is calculated during toe-off plus one sample of the contralateral leg.

Data are provided per walking speed condition, for each adult separately. Data of every single stride of both legs is presented. In addition, for each adult the averaged gait pattern is presented, calculated as the average parameter over all valid individual steps. Moreover data is presented and visualized per gender and age group (18-29 years, 30-39 years, 40-49 years, 50-59 years, 60-69 years and ≥70 years).

## Limitations

In four subjects, the data from at least one speed condition was excluded because of practical issues (i.e. force plate errors) or settings (i.e. a minimum of five correct hits on the force plate is required to be included in the analysis). Furthermore, in this study we cannot rule selection bias as mainly fairly vital elderly subjects participated in the study. This may be caused by the inclusion criteria (e.g., no musculoskeletal diseases) and because the recruitment was through advertising which particularly attracts vital elderly subjects. Moreover, the oldest age group was set at 70years and older. A further division into 70-79 years and 80 years and older would provide even more insight into the walking pattern of the elderly. Finally, data on muscle activation (ectromyography, (EMG)) had not been collected which would further enrich the gait database.

## Ethics Statements

Written informed consent was obtained from all adults prior to participation. The research was carried out in accordance with the Declaration of Helsinki, and the protocol was approved by the local Medical Ethical committee of the MUMC+ (‘Reference gait data for healthy subjects: CAREN-based gait analysis” NL61229.068.17 / METC172015).

## CRediT authorship contribution statement

**Rachel Senden:** Conceptualization, Methodology, Validation, Formal analysis, Investigation, Resources, Writing – original draft, Writing – review & editing, Visualization, Project administration. **Rik Marcellis:** Conceptualization, Methodology, Validation, Formal analysis, Investigation, Resources, Writing – review & editing, Project administration. **Paul Willems:** Software, Validation, Writing – review & editing. **Marianne Witlox:** Conceptualization, Methodology, Writing – review & editing. **Kenneth Meijer:** Conceptualization, Methodology, Writing – review & editing.

## Data Availability

Normative (raw and processed) 3D gait data of healthy adults walking at three different speeds on an instrumented treadmill in virtual reality (Reference data) (OSF).
